# Medial deviation of the ureter is a new sign that could predict stone impaction: a pilot study

**DOI:** 10.1007/s11255-023-03744-5

**Published:** 2023-08-22

**Authors:** Mohammed Aziz, Mohammed Abunayan, Mohammed El Shazly, Baher Salman, Mohammad Habous, Raed Almannie

**Affiliations:** 1 Urology Department, Menoufia University, Shibin El Kom, Egypt; 2 Urology Division, Department of Surgery, College of Medicine, King Saud University, Riyadh, Saudi Arabia

**Keywords:** Ureter, Stone, Impaction, Medial, Deviation

## Abstract

**Objective:**

To assess the value medial deviation of the ureter at site of ureteric stone as a sign of ureteric stone impaction.

**Patients and methods:**

All cases with medial deviation of the ureter at site of ureteric stones in our department over 4 years were enrolled in this pilot study. All cases were diagnosed with KUB and non-contrast CT (NCCT). Radiological and ureteroscopic findings were recorded.

**Results:**

A total of 32 patients with a single impacted stone in the proximal and middle third of the ureter were included in the study. Medial deviation of the ureter at the site of the stone was detected in the upper third of the ureter in 24 (75%) cases and in the middle third in 8 (25%) cases. There were mucosal polyps and mucosal erythema (inflammatory changes) seen by ureteroscopy in all cases (100%). Ureteroscopy was successfully completed with stone fragmentation in 23 (71.8%) patients: 8 of them needed ureteric catheter and 15 required JJ stent insertion. Failure of ureteroscopy with insertion of JJ stent was done in 5 (15.6%) patients. Removal of the stent and ureteroscopy was done after 4 weeks.

**Conclusion:**

We conclude from this study that medial deviation of the ureter is a new reliable radiological sign of ureteric stone impaction.

## Introduction

Urolithiasis affects 2% of the general population with male to female risk of 3–1. Ureteric stones constitute up to 20% of all urinary stones [1–3]. Many factors affect the selection of treatment modalities of ureteric stones. Stone size, site and shape affect the rate of spontaneous passage. For smaller stones, conservative approach is accepted as an initial management modality as it is likely to pass spontaneously. Delayed intervention as shock wave lithotripsy or endourological intervention is reserved to those who fail to pass the stone [4].

Ureteric stones impaction is known to occur at three narrowing sites: the ureteropelvic junction, ureteric crossing of the iliac vessels, and ureterovesical junction (UVJ) [5]. Ureteric stone impaction had been researched as a factor that may affect the choice and the success of different options of treatment of ureteric stones [6–12]. Many definitions have been used, stones that cause moderate to severe hydronephrosis, stones that stay in position for at least 2 months [11], failure to pass a guidewire proximal to the stone [12], or ureteric edema and polyps formation at site of impaction during ureteroscopy [13].

Non contrast computerized tomography (NCCT) is the imaging study of choice for diagnosis of urinary tract stones with a reported sensitivity up to 97% and specificity of 96% for ureteric stones [14–16]. Up till now, there is no specific preoperative sign in NCCT that can suggest ureteric stone impaction. Periureteric edema may develop secondary to inflammatory reaction by neighboring impacted ureteric stones. Ege and coworkers found a periureteric soft tissue stranding due to edema with frequency rate up to 59% [17].

Up to our knowledge there is no other signs of ureteric stone impaction have been described in the literature (except the persistence of the stone in its site by radiological studies, of course).

In this study, we report a new preoperative radiological sign of ureteric stone impaction which is medial deviation of the ureter at the site of stone. We think the presence of this sign may short-cut the way for intervention in such impacted stone especially if they are small. Furthermore, a difficult ureteroscopy is usually expected, especially with voluminous stones.

## Patients and methods

After a period of observation on medial deviation of the ureter at the site of the stone associated with stone impaction and difficult ureteroscopy, we conducted this pilot study.

Inclusion criteria were the following: diagnosis of impacted stone ureter either radiographically (stone in place by NCCT for more than 4 weeks despite MET in stones eligible for medical treatment), or during ureteroscopy for larger stones (> 10 mm) which are eligible for primary intervention. We adopted the concepts of either failure of passing the guide wire proximal to stone ureteroscopy (by a senior endoscopist) or the presence of severe mucosal edema and polyps at site of stone to be the signs of impaction.

We excluded any other possible causes of medial displacement of the ureter like retroperitoneal fibrosis, lymphadenopathy, retrocaval ureter, iliac artery aneurysm, bladder diverticula and pelvic lipomatosis. Any patient with a previous abdominal or pelvic open or laparoscopic surgery were also excluded for fear of post operative adhesions that can cause the deviation. We also excluded patients with previous ureteroscopy interventions.

We recorded clinical findings on presentation, Computerized tomography (CT) finding and ureteroscopy findings in all patients.

After obtaining local ethical committee approval, patients agreed to join the study signed an informed consent. The study was conducted over 4 years from January 2011 to January 2015.

**Fig. 1:**
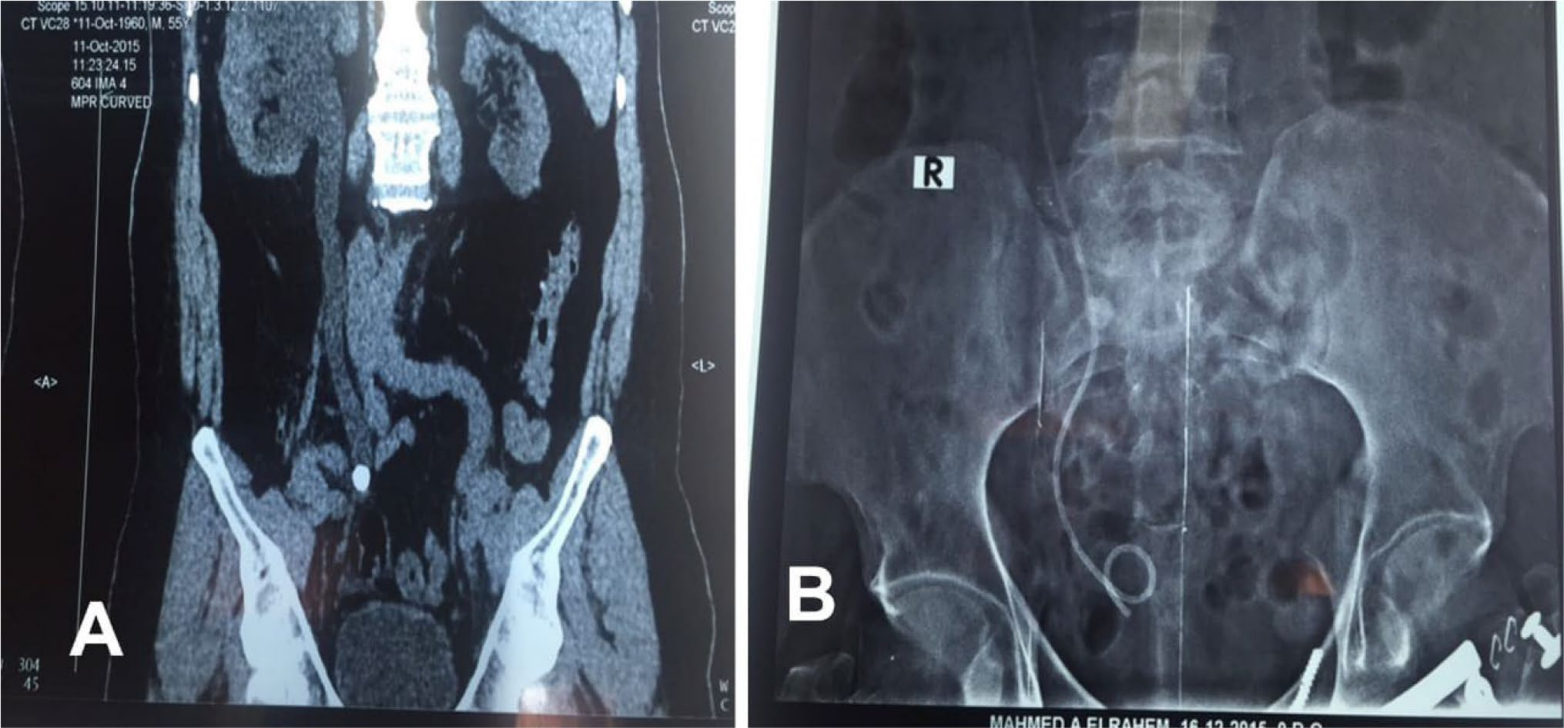
A case of impacted middle third right ureteric radio-opaque stone managed by JJ stent insertion followed by ureteroscopy. NCCT, coronal reformatted image shows medial deviation of the ureter at middle third right ureteric stone. KUB showed right JJ stent and radiopaque middle third right ureteric stone deviated medial to the right sacroiliac joint

All cases were diagnosed preoperatively by plain x ray (KUB) and non-contrast CT, Ultrasonography was not preformed. In KUB, medial deviation was considered when the radio-opaque shadow of the stone is deviated medially to over the body of the lumbar vertebrae in the upper ureter or medial to sacroiliac joint in the middle ureter (Fig. 1b).

**Fig. 2:**
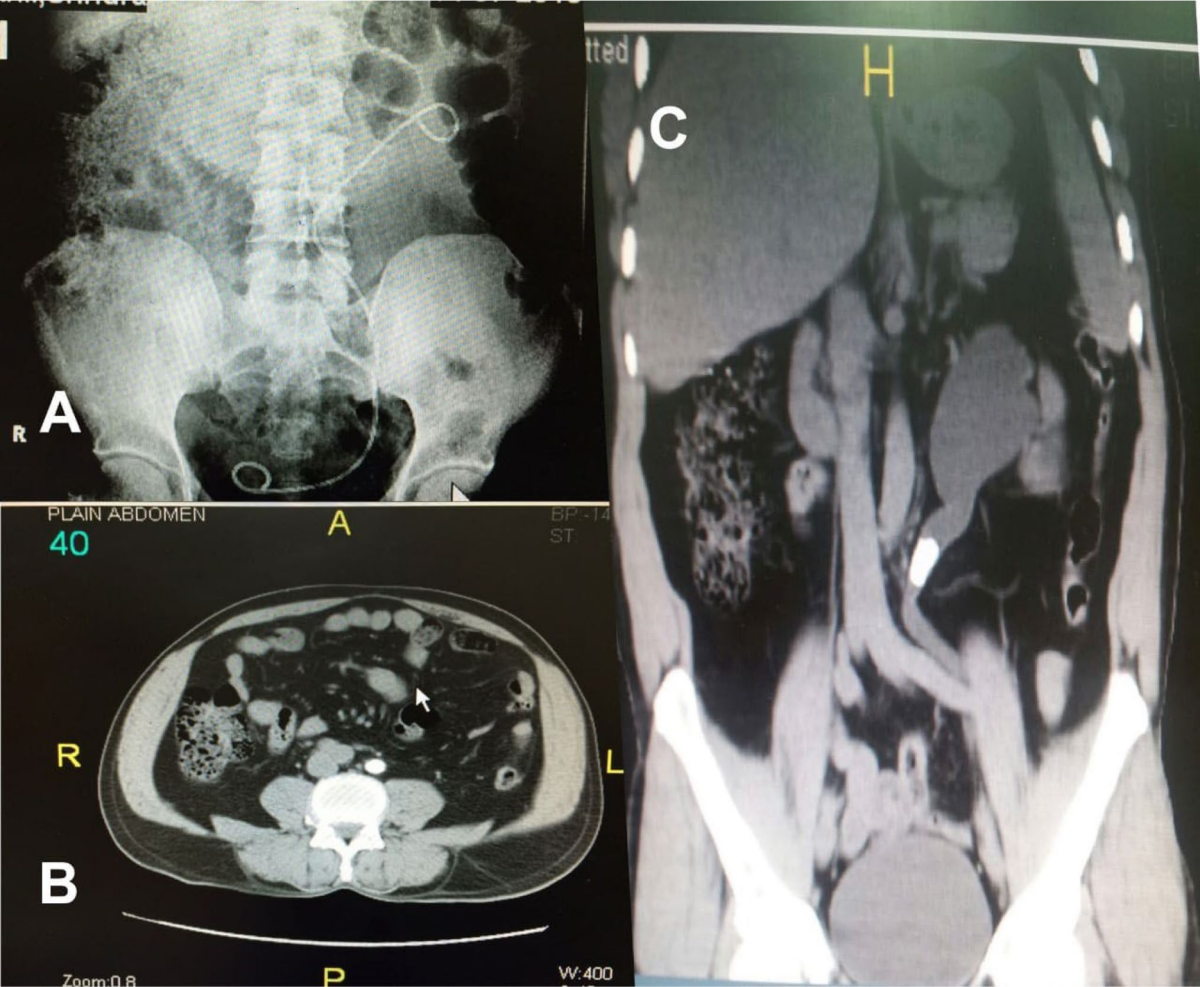
A case of impacted upper third left ureteric radiolucent stone managed by JJ stent insertion followed by ureteroscopy. KUB shows deviation of the jj and ureter at the site of the stone. NCCT, axial cut shows the stone deviated medially anterior to L4 lumbar vertebra. NCCT, coronal reformatted image shows deviation of the ureter at site of stone impaction medially

In NCCT, medial deviation was considered when the ureteric stone was seen anterior to lumbar vertebral body in axial cuts (Fig. 2b) and the ureter bends medially in coronal reformatted image (Fig. 2c) and the ureter lies medial to sacroiliac joint in the middle ureteric stone.

Ureteroscopy disintegration of stones using rigid ureteroscopy with holmium laser using 360 um fibers. Cases with failed ureteroscopy were managed either with JJ stent insertion or percutaneous nephrostomy insertion and antegrade stenting followed by ureteroscopy after 4 weeks.

## Results

Forty three patients met the inclusion criteria. 30 males and 13 females. Of them we found 32 patients 74% to have the medial deviation at site of obstruction radiologically while in the rest 11 patients, there was no medial displacement of the ureter despite the impaction of the stone. Of the 32 patients with positive medial deviation sign, 20 were males and 12 were females. The mean age was 36.4 ± 9.2 years. Obstruction was on the right side in 18 patients (56.3%) and on the left side in 14 (43.7%). Twenty six (81.2%) patients had moderate hydronephrosis (grade 2) and 6 patients (18.8%) had severe hydronephrosis (grade 3). Twenty two patients (68.8%) had stone ≥ 10 mm and 10 patients (32.2%) had stone < 10 mm (Table 1).

**Table 1:** Patients and stone characteristics

Patient gender No. (%)	
Male	20 (62.5%)
Female	12 (37.5%)
Side affected No. (%)	
Right	18 (56.3%)
Left	14 (43.7%)
Degree of hydronephrosis No. (%)	
Moderate (grade II)	26 (81.2%)
Marked (grade III)	6 (18.8%)
Stone size No. (%)	
≥ 10 mm	22 (68.8%)
< 10 mm	10 (31.2%)
Site of stone impaction	
Upper third	24 (75%)
Middle third	8 (25%)
Type of stone	
Radio-opaque	27 (84.4%)
Radiolucent	5 (15.6%)

Twenty-seven (84.4%) patients presented with persistent renal pain not responding to pain medications and 5 patients (15.6%) presented with fever and renal pain (pyelonephritis). Twenty five (78%) patients were on medical expulsive therapy before admission for a period of more than 14 days.

Medial deviation of the ureter at the site of the stone was detected in the upper third of the ureter in 24 (75%) cases and in the middle third in eight (25%) cases. Periureteric stranding at site of stone was detected in five cases only by CT. In all cases (100%) with the medial deviation sign, ureteroscopy showed mucosal polyps and mucosal erythema (inflammatory changes).

In only 3 (9.4%) cases nitinol guide wire with hydrophilic tip passed the stones. Hydrophilic guide wire (Terumo®, Tokyo, Japan) was needed to bypass the stone in 18 (56.2%) cases, and stone disintegration was needed to pass the guide wire under direct vision with ureteroscopy in seven (21.9%) cases. Failed retrograde access was encountered in four (12.5%) patients where percutaneous nephrostomy tube (PCN) was inserted using ultrasound guidance, and antegrade JJ insertion. redo-ureteroscopy was done after 4 weeks.

Out of the 28 cases with guide wire passage, ureteroscopy was successfully completed with stone fragmentation in 23 patients: 8 of them needed ureteric catheter and 15 required JJ stent insertion. Failure of ureteroscopy with insertion of JJ stent was done in five patients. Removal of the stent and ureteroscopy was done after 4 weeks. There were mild ureteric mucosal injuries in three (9.4%) cases that needed JJ insertion. Two cases developed fever after ureteroscopy and JJ insertion that was controlled with antibiotics and antipyretics Table 2. Ureteric stricture formation was encountered in 2 (6.2%) cases after 3 and 7 months postoperatively. Successful management of ureteric strictures in both cases with balloon dilatation was done.

**Table 2:** Ureteroscopy findings

Mucosal edema or polyps	
Present	32 (100%)
Absent	0 (0%)
Guide wire passage (in the 32 patients with medial deviation of the ureter)	
Regular guide wire	3 (9.4%)
Hydrophilic guide wire	18 (56.2%)
Stone disintegration needed before passage of wire	7 (21.9%)
Antegrade JJ insertion	4 (12.5%)

## Discussion

Ureteric stone impaction had been investigated as an important factor that affects the choice and success of different options of treatment of ureteric stones [11, 12]. Moreover, several reports studied the risk of ureteric stricture formation after ureteric stone impaction. Incidence range of 14–24% was reported in different studies [18–20]. This makes radiological diagnosis of ureteric stone impaction crucial to determine the appropriate management modality and to avoid the of complications of long standing impaction as ureteric stricture or impairment of renal function. To predict stone impaction others studies has researched other radiological parameters such as Stone size, Hounsfield unit (HU) of the stone and ureteral wall thickness, they only found that Ureteral wall thickness can predict stone impaction^.^[21, 22]. While others reported that increase of HU has some role in predicting stone impaction [23]. Diagnosis of impaction is usually confirmed intraoperatively when ureteroscopy detects ureteric mucosal polyps formation around stone, edema or distal ureteric tightness [24], with failure or difficulty to pass guidewire or JJ stent proximal to the impacted stones. Few radiological signs can anticipate impaction preoperatively, as hydronephrosis and periureteric soft tissue stranding at site of impaction [17]. In this study, we report this new sign of medial deviation of the ureter at site of stone as a new radiological sign of ureteric stone impaction. In which, the ureter at the site of the impacted stone is acutely deviated medial to the tips of the transverse processes of the vertebrae in the upper third of the ureter or medial to the line of the sacroiliac joint in the middle third. In this study, all cases with medial deviation sign were associated with ureteroscopic signs of impaction which makes this sign a reliable sign to predict ureteric stone impaction.

In our research, we observed a consistent association between cases exhibiting signs of medial deviation and the presence of hydronephrosis as it was all cases hydronephrosis was present. However we cannot definitively conclude that this particular sign is reliable in the absence of hydronephrosis.

Medial deviation can be explained by the effect of forceful peristalsis against the impacted stone trying to expel it. This leads to medial deviation of the ureter as it is related medially to the extraperitoneal potential space and laterally the ureter is supported by psoas major muscle.

### Limitations

The limitations of our study include the relatively small sample size which might limit the generalizability of the findings. The expertise of the physician is essential as it can significantly influence the accuracy and reliability of the radiological and intraoperative findings. The study is a single-center, external validation of the proposed medial deviation sign is necessary in a larger, multicenter study to assess its reliability across different settings.

## Conclusions

Medial deviation of the ureter is a new reliable radiological sign of ureteric stone impaction which can help to avoid intraoperative difficulties and complications. We recommend a senior endoscopist to deal with cases with such sign.
